# Supplementary material for: Adrenomedullin-CALCRL axis controls relapse-initiating drug tolerant acute myeloid leukemia cells
Source: Nat Commun. 2021 Jan 18;12:422. doi: 10.1038/s41467-020-20717-9 (PMC7813857; doi:10.1038/s41467-020-20717-9)
Supplement: Supplementary file 1 — Supplementary information [file 41467_2020_20717_MOESM1_ESM.pdf]

Supplementary Information

Adrenomedullin-CALCRL Axis Controls Relapse-Initiating Drug Tolerant Acute Myeloid Leukemia Cells

Larrue *et al.*

Table of Contents :

- Supplementary Tables 1 - 5
- Supplementary Figures 1-9

Supplementary Tables

Hackl_Relpase_UP						TCGA_Poor_Prognosis		Eppert_LSC_vs_HSC_UP	
CFH	HSPG2	FAH	EPDR1	AEBP1	MYO18A	CFH	IL1RL1	FLJ14213	ETS2
IRX3	ZEB1	ZNRF1	ENO2	TMEM92	IP6K2	IRX3	SCHIP1	HS2ST1	TRPV1
ZNF521	IL1RL1	TSC22D1	C1QTNF3	CEL	AIG1	ZNF521	LRP5	LCT	IKBKB
PTPN14	SCHIP1	GPSM2	LZTS2	TP53INP1	VAT1	PTPN14	USP53	SMYD3	DNAJC4
TM4SF1	ADCY6	LASS4	RALGDS	GLMN	STARD10	TM4SF1	SERPINH1	PTGFR	TAF15
LAPTM4B	LRP5	CYP2S1	ZNF221	ZNF431	HKR1	LAPTM4B	APBB2	ALS2CL	ARL3
CEP70	USP53	TMEM67	TXNRD3	ZNF219	MTHFD1L	CEP70	RBPMS	PRKX	NOTCH3
MYO1B	SERPINH1	ATP1B1	TRIM2	ETV5	DIXDC1	MYO1B	CACNB3	STARD13	PITPNB
SCN9A	APBB2	ALDH5A1	PHKA1	ZSCAN18	DOCK6	SCN9A	ISYNA1	MDFI	NR6A1
TFPI	RBPMS	TNFRSF4	CENPV	FLJ44606	ZNF844	TFPI	ZNF660	PNPLA4	HMG20A
CAV1	CACNB3	C20orf54	ZNF8	HOXB3	AACS	CAV1	EDA2R	CCDC121	ITGB1BP1
SOCS2	ISYNA1	ZNF543	GGT5	ETS2	TTC28	SOCS2	NGFRAP1	GLI2	SS18
SEPP1	ZNF660	SPARC	GNA15	ZNF141	FAM174B	SEPP1	OCIAD2	MPI	TRAF3IP2
FAM38B	EDA2R	GUCY1B3	CD82	CCDC125	C20orf203	FAM38B	MED12L	LOC63920	RTN2
NEGR1	NGFRAP1	IL17RE	KIAA1522	NRSN2	FAHD2B	NEGR1	BCL11A	CALCRL	PPP2CB
MACC1	OCIAD2	CSNK1E	PLAGL1	GCLM	ZNF174	MACC1	GPR56	SLC7A6	ITGA6
CALCRL	MED12L	STARD9	PLA2G6	TBXA2R	SLC38A1	CALCRL		DPY19L4	ARPP-19
MMP2	MDFI	TTC7B	TWISTNB	NFATC2	FAM160B1	MMP2		MRPS14	NPEPPS
RHOBTB3	BCL11A	SLC2A5	ZC4H2	RCN1	ZNF195	RHOBTB3		DOCK1	ABCG1
IL1R1	FZD6	CTTNBP2NL	ACAD11	ZNF35	PALM	IL1R1		TSGA14	KIAA1107
DOCK1	GPR56	NOV	AHDC1	ALS2CR8	LPCAT4	MBOAT2		PEX12	GOSR1
MBOAT2	MFGE8	RBMS2	VN1R1	ANKRD13B	AP1S1	MLLT11		ATP1B1	SLC25A32
MLLT11	PLXNB1	ITGB8	TRIM52	ZNF625	CAMLG	CAV2		NDFIP1	PAFAH1B1
CAV2	CD59	TMSB15B	IFITM1	AMOTL1	AK3	GAS2		FMO4	UBE2Z
GAS2	ZNF610	ZNF334	KANK2	B3GNT5	PNMA1	AKR1C3		DUS4L	WAPAL
AKR1C3	NUB1	ZNF84	GPX7	C9orf174	CD47	TOX		CD200	DLG4
TOX	GIPC3	ARHGAP23	PTMS	ZBED3	DOC2A	CYTL1		TFPT	PHTF1
CYTL1	RHOH	ZNF254	CBX2	DPEP1	SOCS5	CRIP2		SLC9A7	LRRC61
CRIP2	FNBP1L	SUPT3H	CCNB1IP1	KAT2A	IFT140	PCDHB14		ZNF226	ANGPT2
PCDHB14	FSCN1	ZNF177	SERF1A	NKX2-3	KLHL3	GRB10		NPAL2	ADCY6
GRB10	ME1	TREML2	ATP8B2	STOML3	HSF5	RBFOX2		ZNF193	TCEAL1
RBFOX2	GPR125	TP53BP1	LPIN1	GDF11	ACTR5	IGFBP2		MAP7	MON2
IGFBP2	KRT8	CAPN11	TANC1	C11orf80	HPS4	DBN1		ARMC7	SUPT7L
DBN1	ICA1	PCDHB13	GPSM1	ZNF785	PRR4	ITGA2		TBC1D2B	PPP2R5D
ITGA2	LOC646851	TMEM220	PGBD1	CYTH2	RUNX1	SPNS2		MTMR9	PMF1
SPNS2	PTGR1	MAPKBP1	HIST1H2AK	HIST1H4E	ASNS	CMBL		KLHL20	CMAH
CMBL	ZNF382	MMP15	ZSCAN2	ZNF256	GPR89A	PIK3C2B		PAQR6	
PIK3C2B	CFHR1	CLSTN3	GOLGA6L9	FAM171A2	LUZP1	VANGL1		ASS1	
VANGL1	ABI2	RHPN1	DOCK7	TSPYL4	SYNJ2BP	COL6A1		ARMCX5	
COL6A1	SCARA3	C19orf77	MARVELD1	LEPR	ATP5S	MAP1A		NUDT13	
MAP1A	NEDD4	ANKRD28	SLC48A1	SCARB1	LEPREL2	FAM171B		PRKAG2	
FAM171B	ID1	PDE6G	ATP2C1	CXXC5	PPIP5K1	GATA2		INPP5B	
GATA2	KIAA1147	PVRL2	RPAP2	CCDC102A	ADAT2	MYO5C		PTCD2	
MYO5C	ZDHHC9	SNAI1	GOLGA6A	CHRFAM7A	LRP5L	ERG		CDC42BPB	
ERG	SLA2	ZNF273	ZNF10	ZNF669	UBE2E1	WASF1		SYNJ2BP	
WASF1	NET1	SLC16A9	ENPP4	CCDC14	GADD45A	LOC401097		TMF1	
LOC401097	TIE1	B3GALNT1	GATSL1	ZNF768		LAMB2		PURA	
LAMB2	ADAMTS14	STAP1	TPM2	PRICKLE4		KRT18		RALB	
KRT18	TMEM163	CACNA2D4	PHC1	SLC25A36		FADS2		IMPACT	
FADS2	TPD52	MYLK4	C17orf48	PRSS21		GRIK5		EXDL2	
GRIK5	COL24A1	CA8	SH3RF3	FASN		TSPAN2		RAD51L3	
TSPAN2	GPR114	STK33	USP54	HERC2		IRX5		CRKRS	
IRX5	STXBP5	KCNH8	TNFRSF18	PPAPDC1B		RAB38		VPS41	
RAB38	SPRY2	ABCA2	SLC24A1	SEC31B		ERVFRDE1		THAP9	
ANGPT2	ZNF138	UNC13B	ERV3	FGGY		KCNK17		RAD50	
ERVFRDE1	LRRC16A	THBS3	PPM1K	MMP19		MYCN		DSCR3	
KCNK17	ZDHHC15	IFT81	GRAMD1C	ZSCAN30		ZNF711		FAM119B	
MYCN	CABLES1	TGIF2	RTKN	AKR1C1		MSI2		DEDD	
ZNF711	SLC17A9	FADS3	SLC43A1	HIST1H2BF		CD9		CSTF2T	
MSI2	WBP5	LRBA	ATAT1	TCTEX1D2		ARHGEF17		BAHD1	
CD9	TEC	ZNF514	ZBTB26	ZNF251		IKZF2		MGRN1	
ARHGEF17	OR2AK2	RAB13	ZNF577	WDR31		SERPINE1		RER1	
IKZF2	C1orf228	PDLIM1	DEXI	TFB2M		SHANK3		ZBTB39	
SERPINE1	SVIP	GSTM2	ZNF512	LDOC1L		IGSF10		B3GNT1	
SHANK3	PPFIBP1	GPX3	PAFAH1B3	TSPAN3		STON2		PRDX3	
IGSF10	PPT2	FAM117A	BTBD3	NIPA1		STXBP1		AMACR	
STON2	YES1	AUTS2	ANKRD36B	RASSF8		PKD2		ANGPT1	
STXBP1	MEX3B	DUSP14	WDR60	LUC7L3		TARP		SCARB2	
PKD2	TMEM178	SPRY1	TMEM57	ZNF529		OR2L2		APPBP2	
TARP	FAIM	ZNF709	EBPL	ZNF620		EHD2		TRPC2	
OR2L2	NFIX	ZNF69	SSBP2	FAM60A		TOM1L1		WBP4	
EHD2	DDIT4	PPP1R16B	GFI1	SEPT6		SPATS2		AP1S1	
TOM1L1	CDK6	RLTPR	GOLGA6L10	OR52H1		DNM1		MLH3	
SPATS2	C13orf38	COL4A5	COX11	ZMYM3		ATP7B		SFRS1	
DNM1	CASP3	LRRC49	ZNF445	NPM2		NPDC1		HMG20B	
ATP7B	FBXL2	JAG1	CLIC4	PSD3		SPTLC3		PPP2R2A	
NPDC1	RAGE	ZNF345	PLXNA3	TM7SF2		GLP1R		MMRN1	
SPTLC3	SYNJ2	EMID1	PPAP2A	SMPDL3B		TFDP2		KDELR1	
GLP1R	ITGA6	BMP1	TARSL2	STAG3L2		WT1		C1RL	
TFDP2	TRAF5	DPF1	BICD1	ENG		NYNRIN		MEF2C	
WT1	PGM2L1	ZNF749	PTPN7	NLRC3		NDST3		SOCS5	
NYNRIN	PLEKHA5	ALDH18A1	ZNF283	CUBN		MAPK12		RNF41	
NDST3	RALA	TNS1	KCTD3	PCGF3		CPXM1		ACBD4	
MAPK12	BCAT1	PTK7	PRH1	ZNF781		HDGFRP3		RUNX1	
CPXM1	NTRK1	GALNT14	PSPH	STAG3L3		ITGA9		NR1H3	
HDGFRP3	EGLN3	ADA	PYROXD2	TMEM120B		SV2A		TLR3	
ITGA9	BCL7A	CD99	TRAF4	ZSCAN16		PRELID2		AP3S2	
SV2A	ZNF792	SLC7A5	PRTFDC1	IL3RA		GPR146		ACAA2	
PRELID2	PRDM16	KDM5B	C8orf73	ZNF518B		SPP1		SIRT5	
GPR146	ZC3HAV1L	RCL1	SH2D2A	SNX33		DPY19L2		NSMAF	
SPP1	ATP9A	KIAA1549	FAN1	EPT1		CLDN12		MAPRE2	
DPY19L2	DCUN1D3	FOXC1	ZNF608	C11orf63		KIAA0748		STK3	
CLDN12	SLC10A5	H1F0	GNB3	OBSL1		DNMT3B		TBKBP1	
KIAA0748	F2RL1	AGPAT5	PDCL	LRRC61		F2R		DEAF1	
DNMT3B	ASAP2	GSDMB	HIST1H2AM	ZCCHC18		SOX4		CYB561	
F2R	SNCA	KIF9	CENPJ	MTSS1L		CDCA7L		TNPO2	
SOX4	TRIP10	SETMAR	GYG2	ZNF850		HSPG2		TDRD3	
CDCA7L	DOK4	TBC1D16	TRIM47	MXRA7		ZEB1		ANGEL1	

Supplementary Table 1. Gene signatures used to identify CALCRL (related to Figure 1)**.**

Module	GENE	CALCRL high																	CALCRL low														TOTAL
NPM1	NPM1	■	■	■	■	■	■	■											■	■												9
Activated signalling	FLT3	■	■	■	■				■	■	■	■	■						■	■	■											12
	KRAS							■			■							■													■	4
	NRAS																				■							■			■	3
	NF1												■	■																		2
	PTPN11								■																				■			2
	CBL																					■										1
	CSF3R																■															1
	KIT																									■						1
	PIGA																								■							1
DNA methylation	DNMT3A	■				■	■		■					■	■				■													7
	TET2		■													■	■			■							■					5
	IDH2				■										■								■			■						4
	IDH1						■																			■		■				3
Tumor suppressors	TP53									■						■						■	■	■								5
	WT1			■								■									■											3
	PHF6		■																													1
Myeloid TF	CEBPA					■											■			■					■	■						5
	GATA2																			■						■						2
	RUNX1									■				■																		2
	MYC	■																														1
Epigenetic modifier	KMT2D			■													■			■		■										4
	ASXL1																	■							■			■				3
	ASXL2							■																						■		2
	EZH2													■	■																	2
	EP300																												■			1
Cohesin complex	SMC3				■														■													2
	STAG2														■		■															2
	RAD21	■																														1
Spliceosome	SRSF2																			■					■		■	■				4
Other	SETBP1																											■		■		2
	CCND2																									■						1
	TOTAL	5	4	5	4	3	3	3	3	3	2	2	2	4	4	2	5	2	4	7	3	3	2	1	4	6	2	5	2	2	2	99

Supplementary Table 2. Mutational status of the patients for cell surface expression of CALCRL (related to Supplementary Figure 1).

No	CALCRL	ADM	CALCRL.median	ADM.median	ADM.CALCRL	CR1	OS	Death	EFS	Event	No	CALCRL	ADM	CALCRL.median	ADM.median	ADM.CALCRL	CR1	OS	Death	EFS	Event
1	175.00	165.00	low	low	lowlow	1	290	0	290	0	103	225.00	160.00	high	low	lowhigh	0	1571	0	0	1
2	210.00	200.00	high	high	highhigh	0	48	1	48	1	104	207.50	180.00	high	low	lowhigh	0	1528	0	0	1
3	195.00	175.00	high	low	lowhigh	0	364	0	0	1	105	225.00	240.00	high	high	highhigh	1	230	0	230	0
4	160.00	180.00	low	low	lowlow	0	368	0	0	1	106	180.00	245.00	high	high	highhigh	1	788	1	455	1
5	220.00	160.00	high	low	lowhigh	0	69	1	0	1	107	165.00	180.00	low	low	lowlow	0	14	1	14	1
6	215.00	260.00	high	high	highhigh	1	387	0	387	0	108	207.50	285.00	high	high	highhigh	1	1340	0	1340	0
8	200.00	200.00	high	high	highhigh	1	327	0	295	1	109	144.00	160.00	low	low	lowlow	0	465	1	0	1
9	187.50	230.00	high	high	highhigh	1	357	0	357	0	110	215.00	185.00	high	low	lowhigh	1	497	1	406	1
11	230.00	245.00	high	high	highhigh	0	25	1	25	1	112	193.75	190.00	high	low	lowhigh	0	14	1	14	1
12	220.00	250.00	high	high	highhigh	0	15	1	15	1	113	242.50	230.00	high	high	highhigh	1	275	1	179	1
13	160.00	150.00	low	low	lowlow	1	469	0	469	0	114	210.00	250.00	high	high	highhigh	1	329	1	147	1
14	237.50	265.00	high	high	highhigh	0	225	1	0	1	115	160.00	225.00	low	high	highlow	0	1365	0	0	1
15	156.50	140.00	low	low	lowlow	1	171	0	167	1	116	215.00	255.00	high	high	highhigh	1	1534	0	111	1
16	210.00	210.00	high	high	highhigh	0	43	1	43	1	117	218.75	235.00	high	high	highhigh	0	433	1	0	1
18	157.50	135.00	low	low	lowlow	1	536	0	536	0	118	165.00	185.00	low	low	lowlow	0	3	1	3	1
19	200.00	230.00	high	high	highhigh	1	544	0	409	1	119	187.50	130.00	high	low	lowhigh	1	1689	0	1689	0
21	183.75	160.00	high	low	lowhigh	0	48	0	0	1	120	187.50	235.00	high	high	highhigh	0	1301	0	0	1
23	175.00	180.00	low	low	lowlow	0	75	0	0	1	121	166.25	125.00	low	low	lowlow	0	310	1	0	1
24	151.75	195.00	low	high	highlow	0	257	1	0	1	122	140.00	165.00	low	low	lowlow	1	1702	0	1702	0
26	170.00	250.00	low	high	highlow	0	431	1	0	1	123	160.00	215.00	low	high	highlow	0	52	1	0	1
27	195.00	215.00	high	high	highhigh	1	559	0	337	1	125	195.00	270.00	high	high	highhigh	0	1840	0	0	1
28	177.50	170.00	low	low	lowlow	1	632	0	545	1	126	185.00	205.00	high	high	highhigh	1	222	1	206	1
29	174.25	135.00	low	low	lowlow	1	97	0	97	0	127	160.00	245.00	low	high	highlow	0	189	1	0	1
30	187.50	205.00	high	high	highhigh	0	132	1	0	1	128	127.50	145.00	low	low	lowlow	1	297	1	129	1
31	225.00	235.00	high	high	highhigh	0	200	1	0	1	129	150.00	150.00	low	low	lowlow	1	1890	0	1890	0
32	182.50	155.00	high	low	lowhigh	1	653	0	653	0	130	130.00	280.00	low	high	highlow	1	482	1	482	1
33	126.25	135.00	low	low	lowlow	1	660	0	648	1	131	140.50	100.00	low	low	lowlow	1	579	1	139	1
34	217.50	235.00	high	high	highhigh	1	340	1	205	1	132	117.00	115.00	low	low	lowlow	0	1634	0	0	1
35	230.00	200.00	high	high	highhigh	0	39	1	39	1	133	170.00	200.00	low	high	highlow	0	394	1	0	1
36	198.75	145.00	high	low	lowhigh	1	543	0	543	0	134	155.00	110.00	low	low	lowlow	1	1619	0	1619	0
37	158.00	125.00	low	low	lowlow	1	234	0	234	0	135	155.00	100.00	low	low	lowlow	0	30	1	30	1
38	145.50	165.00	low	low	lowlow	1	638	0	638	0	136	175.00	215.00	low	high	highlow	0	82	1	0	1
39	167.50	205.00	low	high	highlow	1	736	0	104	1	138	190.00	235.00	high	high	highhigh	0	490	1	0	1
40	157.50	165.00	low	low	lowlow	0	72	1	0	1	139	220.00	225.00	high	high	highhigh	1	330	1	317	1
41	187.50	205.00	high	high	highhigh	0	491	1	0	1	140	215.00	150.00	high	low	lowhigh	0	536	1	0	1
43	206.25	240.00	high	high	highhigh	0	11	1	11	1	141	160.00	235.00	low	high	highlow	1	1596	0	371	1
44	177.50	155.00	low	low	lowlow	1	767	0	243	1	142	135.00	105.00	low	low	lowlow	1	2108	0	1258	1
46	190.00	220.00	high	high	highhigh	0	179	1	0	1	143	187.50	120.00	high	low	lowhigh	0	267	1	0	1
47	225.00	240.00	high	high	highhigh	0	20	1	20	1	144	157.50	105.00	low	low	lowlow	0	199	1	0	1
48	225.00	215.00	high	high	highhigh	0	128	1	0	1	146	147.50	215.00	low	high	highlow	0	43	1	0	1
49	160.00	205.00	low	high	highlow	0	44	1	0	1	147	161.25	175.00	low	low	lowlow	1	540	1	540	1
50	183.75	155.00	high	low	lowhigh	1	719	0	719	0	148	146.25	140.00	low	low	lowlow	1	2221	0	2221	0
51	182.50	245.00	high	high	highhigh	1	799	0	799	0	149	210.00	290.00	high	high	highhigh	1	2051	0	2051	0
52	120.00	145.00	low	low	lowlow	1	881	0	353	1	150	213.75	210.00	high	high	highhigh	0	199	1	0	1
53	172.50	175.00	low	low	lowlow	1	345	1	336	1	152	182.50	200.00	high	high	highhigh	1	1904	0	1904	0
54	190.00	195.00	high	high	highhigh	0	241	1	0	1	153	127.50	115.00	low	low	lowlow	0	21	1	21	1
55	166.25	255.00	low	high	highlow	1	901	0	201	1	154	275.00	210.00	high	high	highhigh	1	1721	0	422	1
56	141.50	130.00	low	low	lowlow	1	933	0	933	0	155	213.75	235.00	high	high	highhigh	0	232	1	0	1
57	194.75	215.00	high	high	highhigh	0	208	0	0	1	156	116.00	180.00	low	low	lowlow	0	2247	0	0	1
58	170.00	145.00	low	low	lowlow	1	579	0	579	0	157	116.75	100.00	low	low	lowlow	1	769	0	769	0
59	170.00	240.00	low	high	highlow	1	503	1	99	1	158	207.50	270.00	high	high	highhigh	0	144	1	0	1
60	150.00	120.00	low	low	lowlow	1	984	0	385	1	159	170.50	115.00	low	low	lowlow	1	309	1	272	1
61	150.00	110.00	low	low	lowlow	1	987	0	987	0	160	200.00	215.00	high	high	highhigh	1	112	1	89	1
62	161.25	150.00	low	low	lowlow	1	71	1	71	1	161	190.00	290.00	high	high	highhigh	0	574	1	0	1
63	150.00	145.00	low	low	lowlow	1	851	0	851	0	162	155.00	265.00	low	high	highlow	1	713	1	278	1
65	210.00	165.00	high	low	lowhigh	1	722	1	722	1	163	177.50	290.00	low	high	highlow	1	202	1	143	1
67	225.00	215.00	high	high	highhigh	0	187	1	0	1	164	158.75	220.00	low	high	highlow	0	2	1	2	1
69	165.00	150.00	low	low	lowlow	1	806	0	806	0	165	137.50	120.00	low	low	lowlow	1	2514	0	421	1
70	212.50	205.00	high	high	highhigh	0	359	1	0	1	166	180.00	175.00	high	low	lowhigh	1	1545	1	1545	1
71	165.00	215.00	low	high	highlow	0	1064	0	0	1	167	135.00	210.00	low	high	highlow	1	1645	0	1645	0
72	160.00	215.00	low	high	highlow	0	909	0	0	1	168	135.00	100.00	low	low	lowlow	1	552	1	548	1
73	148.75	155.00	low	low	lowlow	0	131	1	0	1	169	178.75	145.00	low	low	lowlow	1	122	0	122	0
74	145.50	135.00	low	low	lowlow	1	1200	0	227	1	170	206.25	275.00	high	high	highhigh	0	107	1	0	1
75	197.50	175.00	high	low	lowhigh	1	222	1	200	1	171	210.00	180.00	high	low	lowhigh	NA	536	1	536	1
76	180.00	205.00	high	high	highhigh	1	72	1	72	1	172	123.00	240.00	low	high	highlow	0	2725	0	0	1
77	220.00	205.00	high	high	highhigh	0	26	1	0	1	173	196.25	185.00	high	low	lowhigh	0	104	1	0	1
78	150.00	135.00	low	low	lowlow	1	1349	0	1349	0	174	160.00	200.00	low	high	highlow	1	1939	1	1939	1
79	180.00	185.00	high	low	lowhigh	0	1186	0	0	1	175	160.00	130.00	low	low	lowlow	0	694	1	0	1
80	235.00	170.00	high	low	lowhigh	0	56	1	56	1	176	205.00	205.00	high	high	highhigh	1	166	1	116	1
81	197.50	200.00	high	high	highhigh	1	1336	0	1336	0	178	158.75	240.00	low	high	highlow	1	892	1	367	1
83	110.00	115.00	low	low	lowlow	1	1270	0	1270	0	179	142.50	175.00	low	low	lowlow	1	2167	0	865	1
84	190.00	145.00	high	low	lowhigh	1	1101	0	1101	0	180	160.00	130.00	low	low	lowlow	1	335	1	72	1
85	166.25	120.00	low	low	lowlow	1	351	1	300	1	181	245.00	275.00	high	high	highhigh	0	29	1	29	1
86	177.50	145.00	low	low	lowlow	1	1154	0	1154	0	182	135.00	130.00	low	low	lowlow	1	3435	0	3435	0
87	207.50	185.00	high	low	lowhigh	0	72	1	0	1	184	197.50	265.00	high	high	highhigh	1	433	0	272	1
88	138.50	120.00	low	low	lowlow	1	424	1	370	1	185	206.25	220.00	high	high	highhigh	0	373	0	0	1
89	163.75	205.00	low	high	highlow	1	549	1	224	1	186	215.00	255.00	high	high	highhigh	0	10	1	10	1
90	115.50	110.00	low	low	lowlow	0	368	1	0	1	187	188.75	175.00	high	low	lowhigh	1	352	0	311	1
91	203.75	160.00	high	low	lowhigh	0	243	1	0	1	188	170.00	160.00	low	low	lowlow	1	2952	0	2952	0
92	185.00	260.00	high	high	highhigh	1	1190	1	757	1	189	126.25	200.00	low	high	highlow	0	2475	1	0	1
93	148.75	215.00	low	high	highlow	1	1447	0	642	1	190	162.50	260.00	low	high	highlow	1	3155	1	3060	1
94	175.00	115.00	low	low	lowlow	0	6	1	6	1	191	196.25	215.00	high	high	highhigh	1	75	0	75	0
95	187.50	230.00	high	high	highhigh	0	443	1	0	1	192	126.00	100.00	low	low	lowlow	1	69	0	69	0
96	171.25	225.00	low	high	highlow	1	1456	0	1456	0	193	182.50	215.00	high	high	highhigh	0	14	1	14	1
97	210.00	190.00	high	low	lowhigh	1	843	1	324	1	195	215.00	250.00	high	high	highhigh	0	81	1	0	1
98	197.00	225.00	high	high	highhigh	1	1538	0	288	1	197	180.00	190.00	high	low	lowhigh	1	253	1	141	1
99	182.50	160.00	high	low	lowhigh	0	302	1	0	1	198	145.00	265.00	low	high	highlow	1	392	0	317	1
100	182.50	185.00	high	low	lowhigh	0	769	0	0	1	200	195.00	250.00	high	high	highhigh	1	66	1	66	1
101	195.00	210.00	high	high	highhigh	1	1556	0	1556	0	201	197.50	270.00	high	high	highhigh	1	577	0	213	1
102	200.00	195.00	high	high	highhigh	0	3	1	3	1											

Supplementary Table 3. CALCRL and ADM expression (related to Figure 1)**.** Cohort of 179 intensively treated AML patients. Patients were clustered into 4 groups according to CALCRL and ADM expression (low/low *vs* low/high *vs* high/low *vs* high/high

Eppert_LSC_vs_HSC_UP	Gentles_LSC_UP	Ng_LSC_17	Farge_HIGHOXPHOS		HALLMARK_OXIDATIVE_PHOSPHORYLATION		HACKL_RELAPSE_UP				CALCRL_UP
FLJ14213	CCDC48	GPR56	ABCB10	MAZ	ABCB7	LRPPRC	CFH	TPD52	EPDR1	PRSS21	PBK
HS2ST1	FAIM3	AKR1C3	ACBD3	MED31	ACAA1	MAOB	IRX3	COL24A1	ENO2	FASN	VAT1L
LCT	GIMAP2	CD34	ACSL3	METTL13	ACAA2	MDH1	ZNF521	GPR114	C1QTNF3	HERC2	KIF14
SMYD3	GIMAP7	NGFRAP1	AIF1	MICAL2	ACADM	MDH2	PTPN14	STXBP5	LZTS2	PPAPDC1B	DSCC1
PTGFR	HSPC159	EMP1	AKAP1	MKI67	ACADSB	MFN2	TM4SF1	SPRY2	RALGDS	SEC31B	DLGAP5
ALS2CL	LOC727893	SMIM24	ALDOC	MOGAT3	ACADVL	MGST3	LAPTM4B	ZNF138	ZNF221	FGGY	KIF11
PRKX	MMRN1	SOCS2	ALG1	MRPL12	ACAT1	MRPL11	CEP70	LRRC16A	TXNRD3	MMP19	TYMS
STARD13	SLC38A1	CPXM1	ARHGEF18	MRPS12	ACO2	MRPL15	MYO1B	ZDHHC15	TRIM2	ZSCAN30	GINS2
MDFI	VNN1	CDK6	ARL4D	MRPS34	AFG3L2	MRPL34	SCN9A	CABLES1	PHKA1	AKR1C1	FANCI
PNPLA4	BIRC3	KIAA0125	ATP5G1	MRPS7	AIFM1	MRPL35	TFPI	SLC17A9	CENPV	HIST1H2BF	TOP2A
CCDC121	CD34	DPYSL3	ATP6V1G2-DDX39B	MRTO4	ALAS1	MRPS11	CAV1	WBP5	ZNF8	TCTEX1D2	CEP55
GLI2	EBF3	MMRN1	AURKAIP1	MS4A4A	ALDH6A1	MRPS12	SOCS2	TEC	GGT5	ZNF251	SKA3
MPI	EVI2A	LAPTM4B	B4GALT2	MVK	ATP1B1	MRPS15	SEPP1	OR2AK2	GNA15	WDR31	NCAPG
LOC63920	GIMAP6	ARHGAP22	BCCIP	MYL6	ATP5A1	MRPS22	FAM38B	C1orf228	CD82	TFB2M	SNAR-E
CALCRL	GUCY1A3	NYNRIN	BNIP3	NAT8L	ATP5B	MRPS30	NEGR1	SVIP	KIAA1522	LDOC1L	NDC80
SLC7A6	HOPX	ZBTB46	BRCA2	NCAPG	ATP5C1	MTRF1	MACC1	PPFIBP1	PLAGL1	TSPAN3	CDK1
DPY19L4	ICAM1	DNMT3B	BRMS1	NCOA5	ATP5D	MTRR	CALCRL	PPT2	PLA2G6	NIPA1	NUSAP1
MRPS14	PCDHGC3		BUB3	NDC80	ATP5E	MTX2	MMP2	YES1	TWISTNB	RASSF8	KIF15
DOCK1	PION		C10orf125	NETO2	ATP5F1	NDUFA1	RHOBTB3	MEX3B	ZC4H2	LUC7L3	PLK4
TSGA14	RBPMS		C16orf80	NFYB	ATP5G1	NDUFA2	IL1R1	TMEM178	ACAD11	ZNF529	SNAR-H
PEX12	SETBP1		C16orf91	NOC4L	ATP5G2	NDUFA3	DOCK1	FAIM	AHDC1	ZNF620	MCM10
ATP1B1	SH3BP5		C1orf112	NRARP	ATP5G3	NDUFA4	MBOAT2	NFIX	VN1R1	FAM60A	CENPE
NDFIP1	ABCC2		C6orf223	NUTF2	ATP5H	NDUFA5	MLLT11	DDIT4	TRIM52	SEPT6	HIST1H2BM
FMO4	FBXO21		CALR	NXT2	ATP5I	NDUFA6	CAV2	CDK6	IFITM1	OR52H1	FAM72D
DUS4L	HECA		CBR1	ORMDL2	ATP5J	NDUFA7	GAS2	C13orf38	KANK2	ZMYM3	NCAPH
CD200	HLF		CCSAP	P2RY2	ATP5J2	NDUFA8	AKR1C3	CASP3	GPX7	NPM2	HIST1H2AB
TFPT	LOC100128550		CCT3	PDE4A	ATP5L	NDUFA9	TOX	FBXL2	PTMS	PSD3	SPAG5
SLC9A7	LTB		CD151	PDIA4	ATP5O	NDUFAB1	CYTL1	RAGE	CBX2	TM7SF2	CLSPN
ZNF226	MEF2C		CD320	PI4K2A	ATP6AP1	NDUFB1	CRIP2	SYNJ2	CCNB1IP1	SMPDL3B	CDC6
NPAL2	SLC37A3		CDC45	PIH1D1	ATP6V0B	NDUFB2	PCDHB14	ITGA6	SERF1A	STAG3L2	SNAR-B2
ZNF193	TMEM200A		CDC6	PIM2	ATP6V0C	NDUFB3	GRB10	TRAF5	ATP8B2	ENG	SNAR-B2
MAP7			CDK1	PKMYT1	ATP6V0E1	NDUFB4	RBFOX2	PGM2L1	LPIN1	NLRC3	MELK
ARMC7			CDK2	PLCXD2-AS1	ATP6V1C1	NDUFB5	IGFBP2	PLEKHA5	TANC1	CUBN	CCNA2
TBC1D2B			CERK	PLK2	ATP6V1D	NDUFB6	DBN1	RALA	GPSM1	PCGF3	MAD2L1
MTMR9			CHML	POLE3	ATP6V1E1	NDUFB7	ITGA2	BCAT1	PGBD1	ZNF781	MND1
KLHL20			CHTF8	POLR2A	ATP6V1F	NDUFB8	SPNS2	NTRK1	HIST1H2AK	STAG3L3	CDCA2
PAQR6			CLIC1	POLR2B	ATP6V1G1	NDUFC1	CMBL	EGLN3	ZSCAN2	TMEM120B	ASPM
ASS1			COG8	POLR2C	ATP6V1H	NDUFC2	PIK3C2B	BCL7A	GOLGA6L9	ZSCAN16	BUB1
ARMCX5			COMMD8	POLR3K	BAX	NDUFS1	VANGL1	ZNF792	DOCK7	IL3RA	TK1
NUDT13			COMMD9	POMP	BCKDHA	NDUFS2	COL6A1	PRDM16	MARVELD1	ZNF518B	TTK
PRKAG2			COPB2	POP4	BDH2	NDUFS3	MAP1A	ZC3HAV1L	SLC48A1	SNX33	SPC24
INPP5B			CRELD2	POP7	BRP44L	NDUFS4	FAM171B	ATP9A	ATP2C1	EPT1	CCNB2
PTCD2			CSNK1G1	PRMT7	CASP7	NDUFS6	GATA2	DCUN1D3	RPAP2	C11orf63	FBXO5
CDC42BPB			CXXC1	PSMC3	COX10	NDUFS7	MYO5C	SLC10A5	GOLGA6A	OBSL1	HIST1H1B
SYNJ2BP			DAXX	PSME3	COX11	NDUFS8	ERG	F2RL1	ZNF10	LRRC61	KIF20A
TMF1			DEPDC7	PTBP1	COX15	NDUFV1	WASF1	ASAP2	ENPP4	ZCCHC18	SNAR-C4
PURA			DERL3	PXMP2	COX17	NDUFV2	LOC401097	SNCA	GATSL1	MTSS1L	SNAR-C3
RALB			DGCR8	QRICH1	COX4I1	NNT	LAMB2	TRIP10	TPM2	ZNF850	PLK1
IMPACT			DHX9	RAB35	COX5A	NQO2	KRT18	DOK4	PHC1	MXRA7	NEK2
EXDL2			DNAJA1	RBM19	COX5B	OAT	FADS2	FAH	C17orf48	MYO18A	TPX2
RAD51L3			DNAJB11	RFC3	COX6A1	OGDH	GRIK5	ZNRF1	SH3RF3	IP6K2	HMX2
CRKRS			DNTTIP1	RFC5	COX6B1	OPA1	TSPAN2	TSC22D1	USP54	AIG1	EXO1
VPS41			DPM1	RNF5	COX6C	OXA1L	IRX5	GPSM2	TNFRSF18	VAT1	DEPDC1B
THAP9			DTL	RNPS1	COX7A2	PDHA1	RAB38	LASS4	SLC24A1	STARD10	HMMR
RAD50			DTYMK	RPS26	COX7A2L	PDHB	ANGPT2	CYP2S1	ERV3	HKR1	SKA1
DSCR3			DUS3L	SCD	COX7B	PDHX	ERVFRDE1	TMEM67	PPM1K	MTHFD1L	SGOL1
FAM119B			DUSP23	SDF2L1	COX7C	PDK4	KCNK17	ATP1B1	GRAMD1C	DIXDC1	CASC5
DEDD			DVL2	SEH1L	COX8A	PDP1	MYCN	ALDH5A1	RTKN	DOCK6	SLC38A1
CSTF2T			E2F7	SF3A1	CPT1A	PHB2	ZNF711	TNFRSF4	SLC43A1	ZNF844	BCL2
BAHD1			ECRP	SF3B5	CS	PHYH	MSI2	C20orf54	ATAT1	AACS	MYBL2
MGRN1			EIF4A3	SFXN5	CYB5A	PMPCA	CD9	ZNF543	ZBTB26	TTC28	BRIP1
RER1			EIF4EBP2	SIRT6	CYB5R3	POLR2F	ARHGEF17	SPARC	ZNF577	FAM174B	CENPF
ZBTB39			EIF4G1	SKAP1	CYC1	POR	IKZF2	GUCY1B3	DEXI	C20orf203	GTSE1
B3GNT1			EPX	SKP2	CYCS	PRDX3	SERPINE1	IL17RE	ZNF512	FAHD2B	MTFR2
PRDX3			EXO1	SLC25A1	DECR1	RETSAT	SHANK3	CSNK1E	PAFAH1B3	ZNF174	E2F8
AMACR			FARSA	SLC25A10	DLAT	RHOT1	IGSF10	STARD9	BTBD3	SLC38A1	FEN1
ANGPT1			FASN	SLC25A19	DLD	RHOT2	STON2	TTC7B	ANKRD36B	FAM160B1	CENPK
SCARB2			FBXO16	SLC29A1	DLST	SDHA	STXBP1	SLC2A5	WDR60	ZNF195	HIST1H2AI
APPBP2			FKBPL	SMARCD2	ECH1	SDHB	PKD2	CTTNBP2NL	TMEM57	PALM	CDC20
TRPC2			FPGS	SNRPD1	ECHS1	SDHC	TARP	NOV	EBPL	LPCAT4	HELLS
WBP4			GDF11	SPAG5	ECI1	SDHD	OR2L2	RBMS2	SSBP2	AP1S1	ASF1B
AP1S1			GINS3	SPN	ETFA	SLC25A11	EHD2	ITGB8	GFI1	CAMLG	KIAA0226L
MLH3			GLYR1	SREBF1	ETFB	SLC25A12	TOM1L1	TMSB15B	GOLGA6L10	AK3	WDR76
SFRS1			GNA11	SRSF2	ETFDH	SLC25A20	SPATS2	ZNF334	COX11	PNMA1	CHEK1
HMG20B			GPR183	ST3GAL6-AS1	FDX1	SLC25A3	DNM1	ZNF84	ZNF445	CD47	HIST1H3J
PPP2R2A			GTSE1	STRA13	FH	SLC25A4	ATP7B	ARHGAP23	CLIC4	DOC2A	RAD51AP1
MMRN1			H3F3B	SUPT4H1	FXN	SLC25A5	NPDC1	ZNF254	PLXNA3	SOCS5	MPO
KDELR1			HIST1H2BG	SWSAP1	GLUD1	SLC25A6	SPTLC3	SUPT3H	PPAP2A	IFT140	CCNB1
C1RL			HIST1H3J	SYNCRIP	GOT2	SUCLA2	GLP1R	ZNF177	TARSL2	KLHL3	RAD51
MEF2C			HIST1H4D	TAC1	GPI	SUCLG1	TFDP2	TREML2	BICD1	HSF5	SNAR-D
SOCS5			HIST2H2AA4	TECR	GPX4	SUPV3L1	WT1	TP53BP1	PTPN7	ACTR5	CHRNA5
RNF41			HIST2H3D	THOC6	GRPEL1	SURF1	NYNRIN	CAPN11	ZNF283	HPS4	NUF2
ACBD4			HIST2H4B	TIMM13	HADHA	TCIRG1	NDST3	PCDHB13	KCTD3	PRR4	KIF2C
RUNX1			HLA-DMA	TIMM21	HADHB	TIMM10	MAPK12	TMEM220	PRH1	RUNX1	ARHGAP11A
NR1H3			HMGB3P1	TK1	HCCS	TIMM13	CPXM1	MAPKBP1	PSPH	ASNS	SPC25
TLR3			HSP90B1	TLCD1	HSD17B10	TIMM17A	HDGFRP3	MMP15	PYROXD2	GPR89A	TNFRSF19
AP3S2			HSPA5	TMEM8A	HSPA9	TIMM50	ITGA9	CLSTN3	TRAF4	LUZP1	SHCBP1
ACAA2			HSPH1	TNPO2	HTRA2	TIMM8B	SV2A	RHPN1	PRTFDC1	SYNJ2BP	WEE1
SIRT5			IL17RA	TRA2B	IDH1	TIMM9	PRELID2	C19orf77	C8orf73	ATP5S	KIF23
NSMAF			ISG20L2	TREX1	IDH2	TOMM22	GPR146	ANKRD28	SH2D2A	LEPREL2	GINS1
MAPRE2			ISOC1	TRIAP1	IDH3A	TOMM70A	SPP1	PDE6G	FAN1	PPIP5K1	CTNNAL1
STK3			KIF11	TRIM28	IDH3B	UQCR10	DPY19L2	PVRL2	ZNF608	ADAT2	BLM
TBKBP1			KIF20B	TRIP13	IDH3G	UQCR11	CLDN12	SNAI1	GNB3	LRP5L	PSIP1
DEAF1			KIF24	TUBB	IMMT	UQCRB	KIAA0748	ZNF273	PDCL	UBE2E1	CKAP2L
CYB561			KIFC1	TULP3	ISCA1	UQCRC1	DNMT3B	SLC16A9	HIST1H2AM	GADD45A	FAM83D
TNPO2			KITLG	TXNDC17	ISCU	UQCRC2	F2R	B3GALNT1	CENPJ		CCDC34
TDRD3			KLHDC3	TXNIP	LDHA	UQCRFS1	SOX4	STAP1	GYG2		TICRR
ANGEL1			KLK3	TYMS	LDHB	UQCRH	CDCA7L	CACNA2D4	TRIM47		TMEM255A
ETS2			LGR4	UBAP2L		UQCRQ	HSPG2	MYLK4	AEBP1		RRM1
TRPV1			LINC00467	UBE2C		VDAC1	ZEB1	CA8	TMEM92		CCNF
IKBKB			LINC00514	UBL5		VDAC2	IL1RL1	STK33	CEL		DTL
DNAJC4			LMNB1	UHRF1		VDAC3	SCHIP1	KCNH8	TP53INP1		MCM3
TAF15			LOC100129048	UNC93B1			ADCY6	ABCA2	GLMN		CDCA8
ARL3			LOC100289061	UNG			LRP5	UNC13B	ZNF431		TRIP13
NOTCH3			LOC100506165	UTP20			USP53	THBS3	ZNF219		ESCO2
PITPNB			LOC727803	VPS25			SERPINH1	IFT81	ETV5		MCM2
NR6A1			LRRC26	XRCC6BP1			APBB2	TGIF2	ZSCAN18		LOC642934
HMG20A			LRRC49	YDJC			RBPMS	FADS3	FLJ44606		BIRC5
ITGB1BP1			LRRC59	ZBTB40			CACNB3	LRBA	HOXB3		CDC45
SS18			MARCKSL1	ZBTB9			ISYNA1	ZNF514	ETS2		TAF9B
TRAF3IP2				ZWINT			ZNF660	RAB13	ZNF141		INCENP
RTN2							EDA2R	PDLIM1	CCDC125		MKI67
PPP2CB							NGFRAP1	GSTM2	NRSN2		STIL
ITGA6							OCIAD2	GPX3	GCLM		AURKB
ARPP-19							MED12L	FAM117A	TBXA2R		FOXM1
NPEPPS							MDFI	AUTS2	NFATC2		PRIM1
ABCG1							BCL11A	DUSP14	RCN1		HAUS1
KIAA1107							FZD6	SPRY1	ZNF35		KIF18A
GOSR1							GPR56	ZNF709	ALS2CR8		NET1
SLC25A32							MFGE8	ZNF69	ANKRD13B		CENPU
PAFAH1B1							PLXNB1	PPP1R16B	ZNF625		MCM4
UBE2Z							CD59	RLTPR	AMOTL1		ORC6
WAPAL							ZNF610	COL4A5	B3GNT5		TIMELESS
DLG4							NUB1	LRRC49	C9orf174		HIST1H1C
PHTF1							GIPC3	JAG1	ZBED3		MCM7
LRRC61							RHOH	ZNF345	DPEP1		MT1G
ANGPT2							FNBP1L	EMID1	KAT2A		SMC2
ADCY6							FSCN1	BMP1	NKX2-3		HIST1H2AM
TCEAL1							ME1	DPF1	STOML3		OXCT1
MON2							GPR125	ZNF749	GDF11		ZWILCH
SUPT7L							KRT8	ALDH18A1	C11orf80		HIST1H2AE
PPP2R5D							ICA1	TNS1	ZNF785		CIB3
PMF1							LOC646851	PTK7	CYTH2		MCM6
CMAH							PTGR1	GALNT14	HIST1H4E		WDHD1
							ZNF382	ADA	ZNF256		USP1
							CFHR1	CD99	FAM171A2		DEPDC1
							ABI2	SLC7A5	TSPYL4		RFC5
							SCARA3	KDM5B	LEPR		HIST2H2AB
							NEDD4	RCL1	SCARB1		HIST1H2BL
							ID1	KIAA1549	CXXC5		OIP5
							KIAA1147	FOXC1	CCDC102A		POLE2
							ZDHHC9	H1F0	CHRFAM7A		SPIN4
							SLA2	AGPAT5	ZNF669		CHAF1B
							NET1	GSDMB	CCDC14		
							TIE1	KIF9	ZNF768		
							ADAMTS14	SETMAR	PRICKLE4		
							TMEM163	TBC1D16	SLC25A36		

Supplementary Table 4. Gene signatures used for GSEA analyses. (GO_CELL_CYCLE, GO_DNA_REPAIR and GO_DNA_INTEGRITY_CHECKPOINT are not included in this table and are available on GSEA website).

Patient ID	Hospital	FAB	WBC (G/L)	D/R	Karyotype	FLT3-ITD	NPMc
1	TUH	M4	37	D	46, XY	1	1
2	TUH	M1	20	D	46, XY	1	1
3	TUH	M1	7.7	D	46, XY	1	0
4	TUH	M1	68.8	D	46, XY	1	1
5	TUH	M5	247	D	46, XX	1	0
6	TUH	M2	64.9	R	47,XY,inv(1)(p21q44),+8,add(13)(q34),t(16;17)(p12;q12)[8] 47,XY,t(1;6)(p22;q27),del(3)(q26)or t(3;7)(q26;p15), add(7)(p21),+8,t(14;15)(q24;q25)[12]	0	0
7	TUH	M1	48	D	46, XY	0	0
8	TUH	M4	14.7	D	46, XY	1	0
9	TUH	M1	6	D	46,der(X)t(X;X)(q28;q26),del(X)(q13q27),ins(6;?)(q16 or q22;?) or dup(6)(q21)[21]/ 44,X,-X,del(2)(p25p14),add(6)(p25),add(8)(q24),add(19)(p11),-22,+mar[5]/46,XX[6]	0	0
10	TUH	M5	136.9	D	46, XY	1	0
11	TUH	M4	288	D	46, XY	1	0
12	TUH	M4	112.78	D	46,XY	1	1
13	TUH	M1	4	D	46,XY	0	1
14	TUH	M1	38	D	46,XY	1	1
15	TUH	M5	32.1	D	46,XY	1	1
16	TUH	M2	47.8	D	46, XY	0	1
17	TUH	M2	110	D	46,XY	1	0
18	TUH	M1	10	D	46, XY	0	1
19	TUH	M1	30	D	46, XX	0	1
20	TUH	M1	87.99	D	46, XX	1	0
21	TUH	M1	37.6	D	46, XY	1	1
22	TUH	M1	118.6	D	46, XY	1	1
23	TUH	M4	83.5	D	46, XY	0	1
24	TUH	M1	247	D	46, XX	1	0
25	TUH	M5	306.8	D	46, XX	0	1
26	TUH	M1	161	D	46, XX	0	1
27	TUH	M4	207.6	D	46, XX	1	0
28	TUH	M4	59.2	D	46, XY	1	1
29	TUH	M1	263.6	D	46,XX,t(1;6)(p10;q10),inv3(p14q24)<20>	ND	ND
30	TUH	M1	87	D	46,XX,t(6;14)(q21;q32)<2>/46,XX<18>	1	1
31	TUH	M4	73.6	D	46, XX	1	0
32	TUH	M5	12.4	D	46, XY	1	1
33	TUH	M4	54.9	D	46, XX	0	0
34	TUH	M5		D	46, XX	0	0
35	TUH	M1		D			
36	TUH	M4		D			

Supplementary Table 5. Patient characteristics. (Western-Blot, clonogenic assays, LDA)

Supplementary Figures

Supplementary Figure 1

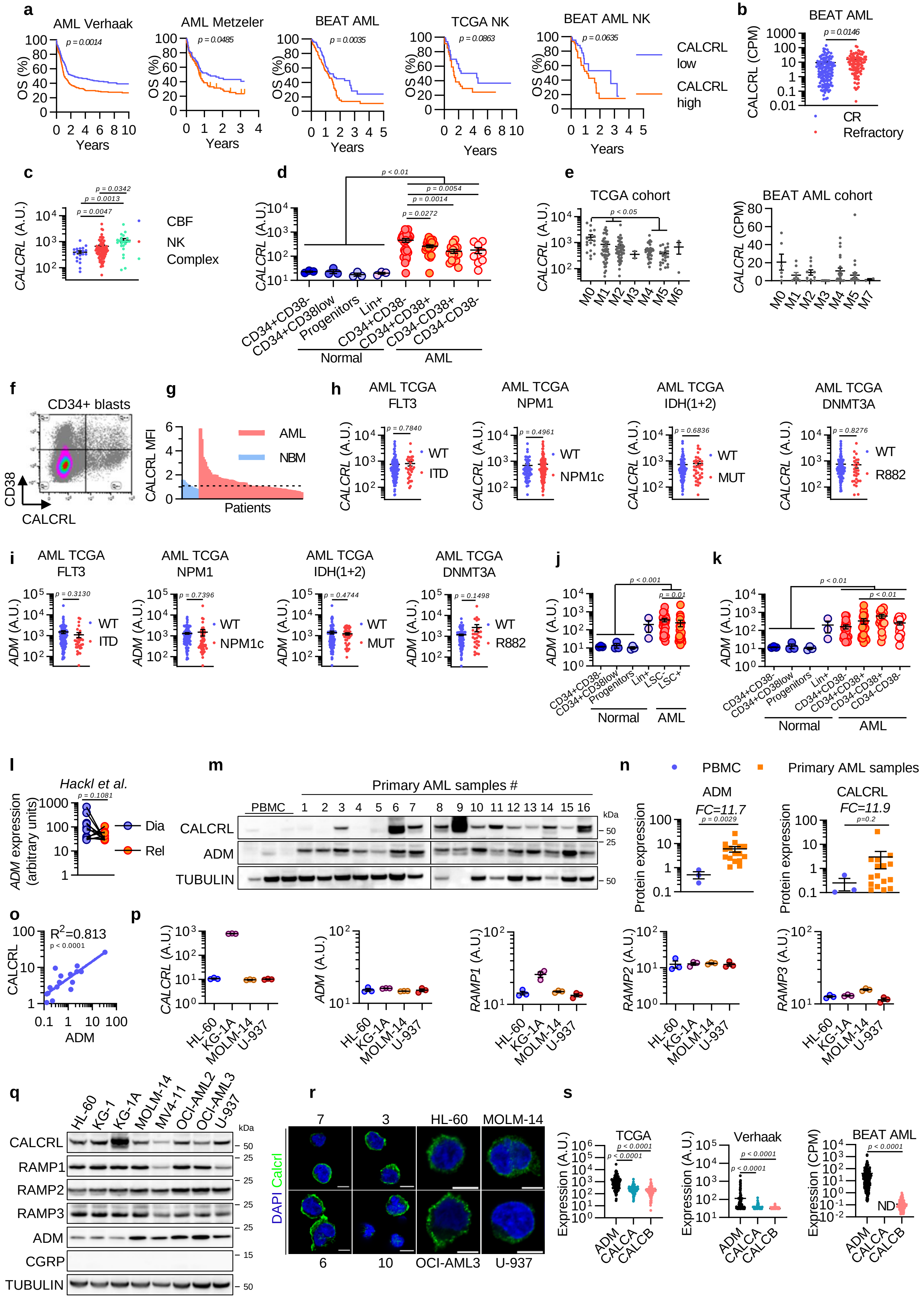

Supplementary Figure 1. Expression of CALCRL and its ligand adrenomedullin and impact on patient outcome in AML

a. Impact of *CALCRL* gene expression on overall survival (Log-rank (Mantel-Cox) test). **b.** *CALCRL* expression (BEAT AML cohort) in patients achieving a complete remission or refractory to chemotherapy (Unpaired t test). **c.** *CALCRL* expression (BEAT AML cohort) in CBF, NK and Complex AML (Unpaired t test). **d.** *CALCRL* gene expression in normal *versus* leukemic CD34+CD38-, CD34+CD38low, progenitors and Lin+ compartments according to Eppert *et al*. (Unpaired t test). **e.** *CALCRL* expression (TCGA and BEAT AML cohorts) according to FAB subtypes (Unpaired t test). **f.** Flow cytometry plot shows CALCRL expression on CD34+ primary AML cells. **g.** Percentage of cells expressing CALCRL was assessed by flow cytometry analysis of normal CD34+ and leukemic cells. **h.** Expression of *CALCRL* according to mutational status (AML TCGA) (Unpaired t test). **i.** Expression of *ADM* according to mutational status (AML TCGA) (Unpaired t test). **j.** *ADM* gene expression in normal compartments CD34+CD38-, CD34+CD38low, progenitors and Lin+ compared with leukemic LSC- and LSC+ compartments (Eppert *et al.*) (Unpaired t test) **k.** *ADM* gene expression in normal *versus* leukemic CD34+CD38-, CD34+CD38low, progenitors and Lin+ compartments (Eppert *et al.*) (Unpaired t test) **l.** *ADM* expression at diagnosis and relapse after intensive chemotherapy (Hackl *et al*.) (Unpaired t test) **m.** Western Blot results showing expression of CALCRL and ADM in normal peripheral blood mononuclear cells (n=3) and in a panel of primary AML samples (n=16). **n.** Quantification of Western Blot (Unpaired t test). **o.** Correlation between CALCRL and ADM protein expression (Sample linear regression). **p.** *CALCRL*, *ADM*, *RAMP1*, *RAMP2* and *RAMP3* gene expression in AML cell lines. **q.** Western Blot results showing expression of CALCRL, RAMP1, RAMP2, RAMP3, ADM and CGRP in a panel of AML cell lines (n=1). **r.** Confocal analysis of the expression of CALCRL (in green) in primary AML samples and AML cell lines. Scale bar: 10 µm. **s.** *ADM*, *CALCA* and *CALCB* expression (TCGA, Verhaak *et al.* and BEAT AML cohorts) (Unpaired t test).

Supplementary Figure 2

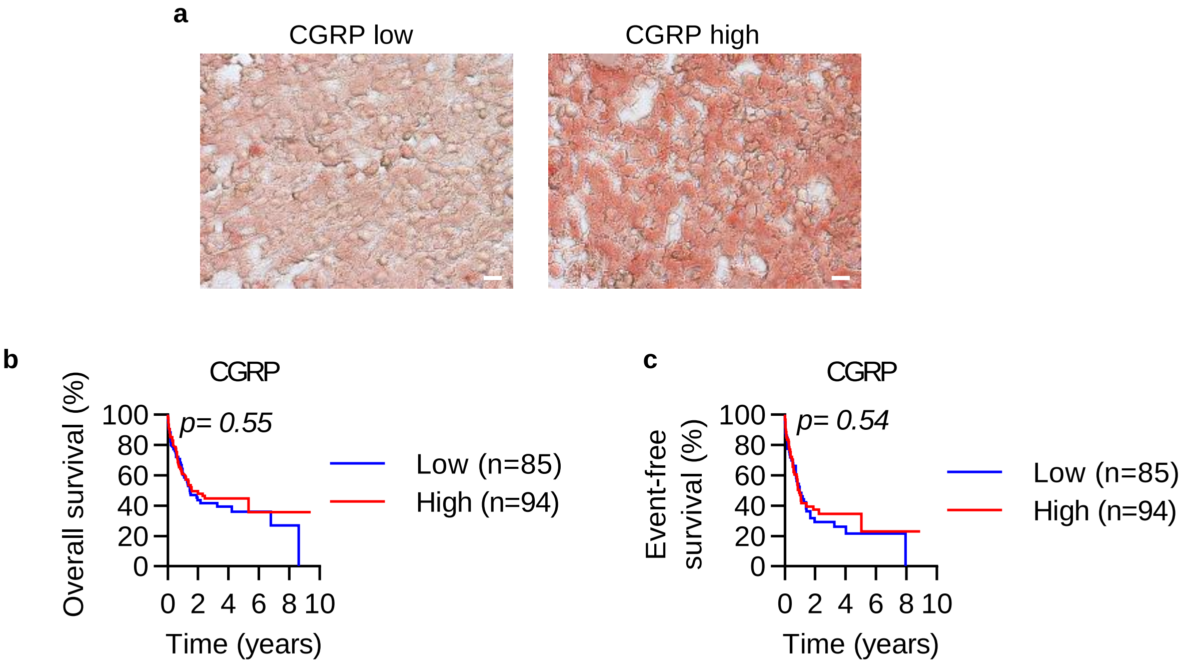

Supplementary Figure 2. Expression of CGRP and impact on patient outcome in AML.
a. Representative IHC micrographs of CGRP expression in pretherapeutic BM from AML patients. Scale bar: 10 µm. **(b)** and **(c)** Overall and Event-Free Survival according to CGRP H-scores. OS and EFS were regressed against CGRP H-scores using univariate or multivariate Cox model. Data were dichotomized for visualization purpose.

Supplementary Figure 3

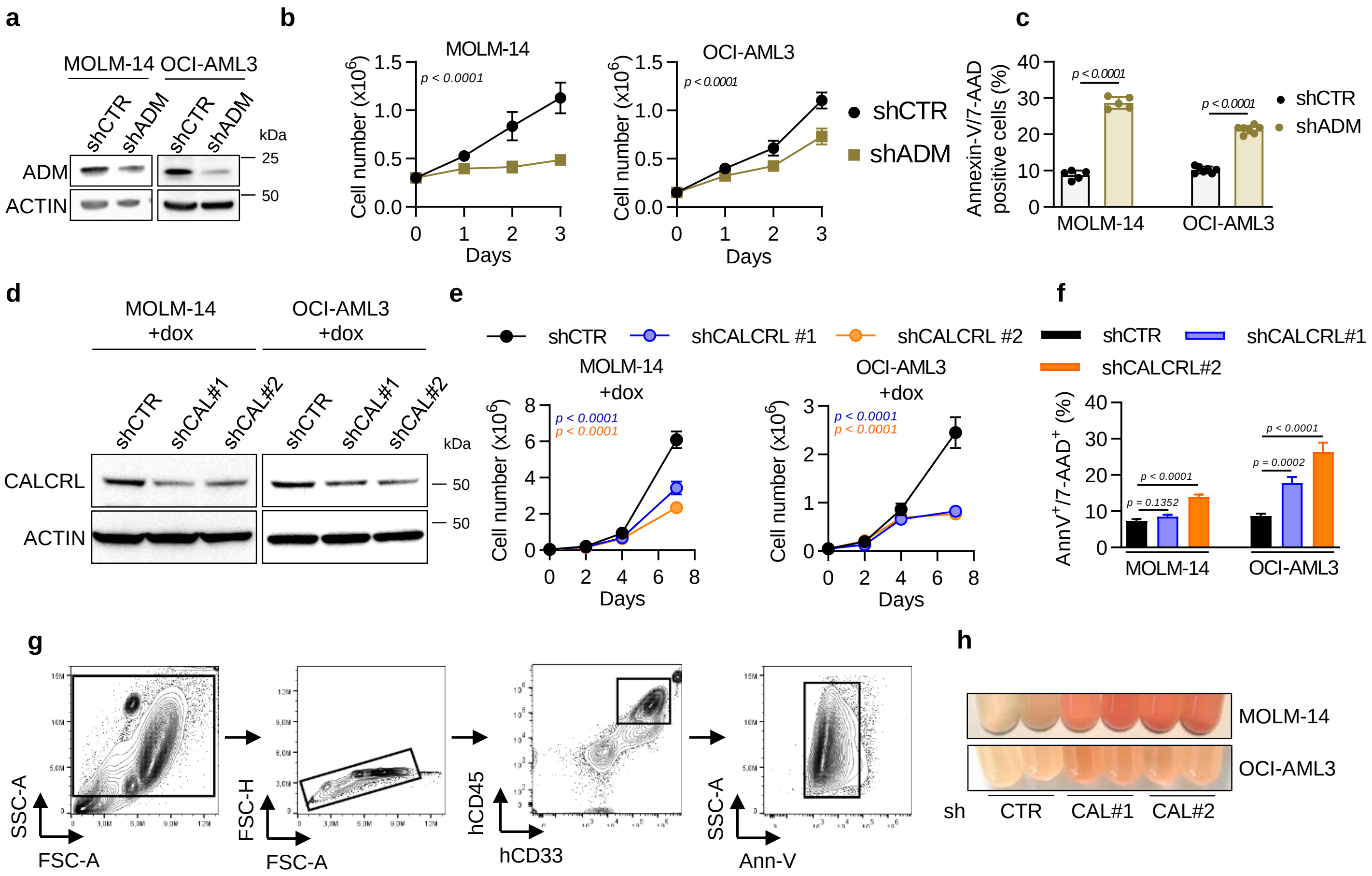

Supplementary Figure 3. Depletion of CALCRL or ADM impairs leukemic cell growth.
a. Western blot results showing expression of ADM and β-ACTIN proteins in MOLM-14 and OCI-AML3 four days after transduction with shADM (n=2). **b.** Graph shows cell number of MOLM-14 or OCI-AML3. Three days after transduction, cells were plated (D0) and cell proliferation was followed using trypan blue exclusion (n=6, Two-way ANOVA). **c.** Graph shows the percentage of Annexin-V+ or 7-AAD+ cells 4 days after cell transduction (n=5, Unpaired t test). **d.** Western blot results showing expression of CALCRL and β-ACTIN proteins in MOLM-14 and OCI-AML3 transduced with inducible shRNAs targeting CALCRL (n=2). Protein expression was assessed 7 days after the induction of shRNA with doxycycline (1 µg/ml). **e.** Graph shows cell number of MOLM-14 or OCI-AML3. Doxycycline was added at D0 and cell proliferation was followed using trypan blue exclusion (n=6, Two-way ANOVA). **f.** Graph shows the percentage of Annexin-V+ or 7-AAD+ cells 7 days after adding doxycycline into cell culture medium (Unpaired t test). **g.** Flow cytometry plots showing the gating strategy used to identify human leukemic cells issued from MOLM-14 or OCI-AML3 xenografts. **h.** Picture of mice bone marrow engrafted with indicated shRNA.

Supplementary Figure 4

a

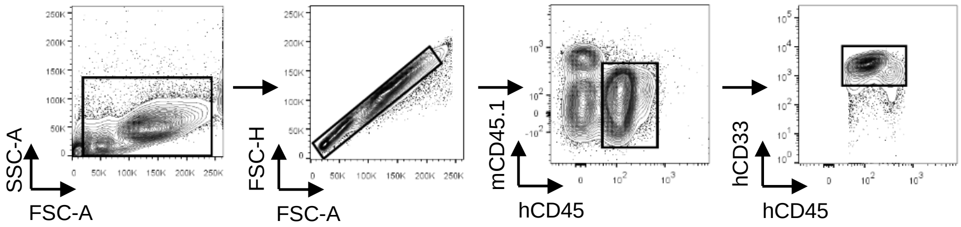

Supplementary Figure 4. Identification of human leukemic cells issued from primary AML xenografts.

a. Flow cytometry plots showing the gating strategy used to identify human leukemic cells issued from primary AML xenografts.

Supplementary Figure 5

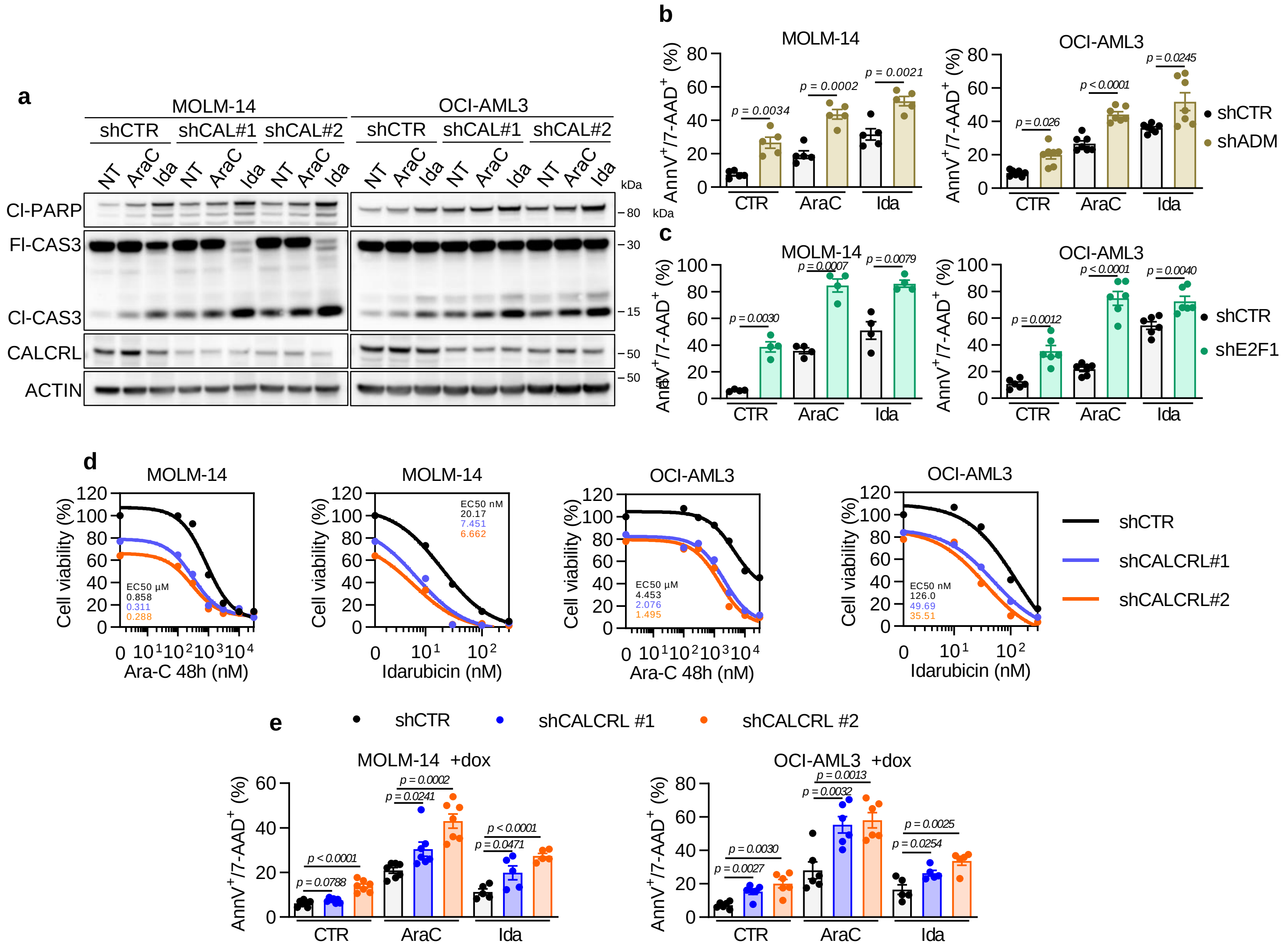

Supplementary Figure 5. Depletion of CALCRL sensitizes to chemotherapeutic drugs *in vitro*
a. Western-Blotting for PARP, CASPASE-3, CALCRL and β-ACTIN (n=1). Cells were treated 24h with AraC (2 µM) or Idarubicin (50 nM). **b-c.** Graph shows the percentage of Annexin-V+ or 7-AAD+ cells. Three days after transduction with shCTR, shADM (**b**) (n=5), or shE2F1 (**c**) (n=7), cells were treated with AraC (2 µM) or Idarubicin (50 nM) for 48h before flow cytometry analysis (Unpaired t test). **d.** Five days after adding doxycycline, MOLM-14 or OCI-AML3 cells expressing inducible shRNAs were treated with AraC or idarubicin for 48h (n=1 in triplicate). Then cell viability was assessed by MTS assay and values were normalized to untreated condition. Curve fit to calculate IC50 was determined by log (inhibitor) *vs.* response (three parameters) test. **e.** Graph shows the percentage of Annexin-V+ or 7-AAD+ cells. Five days after adding doxycycline, MOLM-14 or OCI-AML3 cells expressing inducible shRNAs were treated with AraC (2 µM) or idarubicin (50 nM) for 48h. Then, the percentage of cell death was monitored using Annexin-V+ and 7-AAD+ markers (n=5-7, Unpaired t test).

Supplementary Figure 6

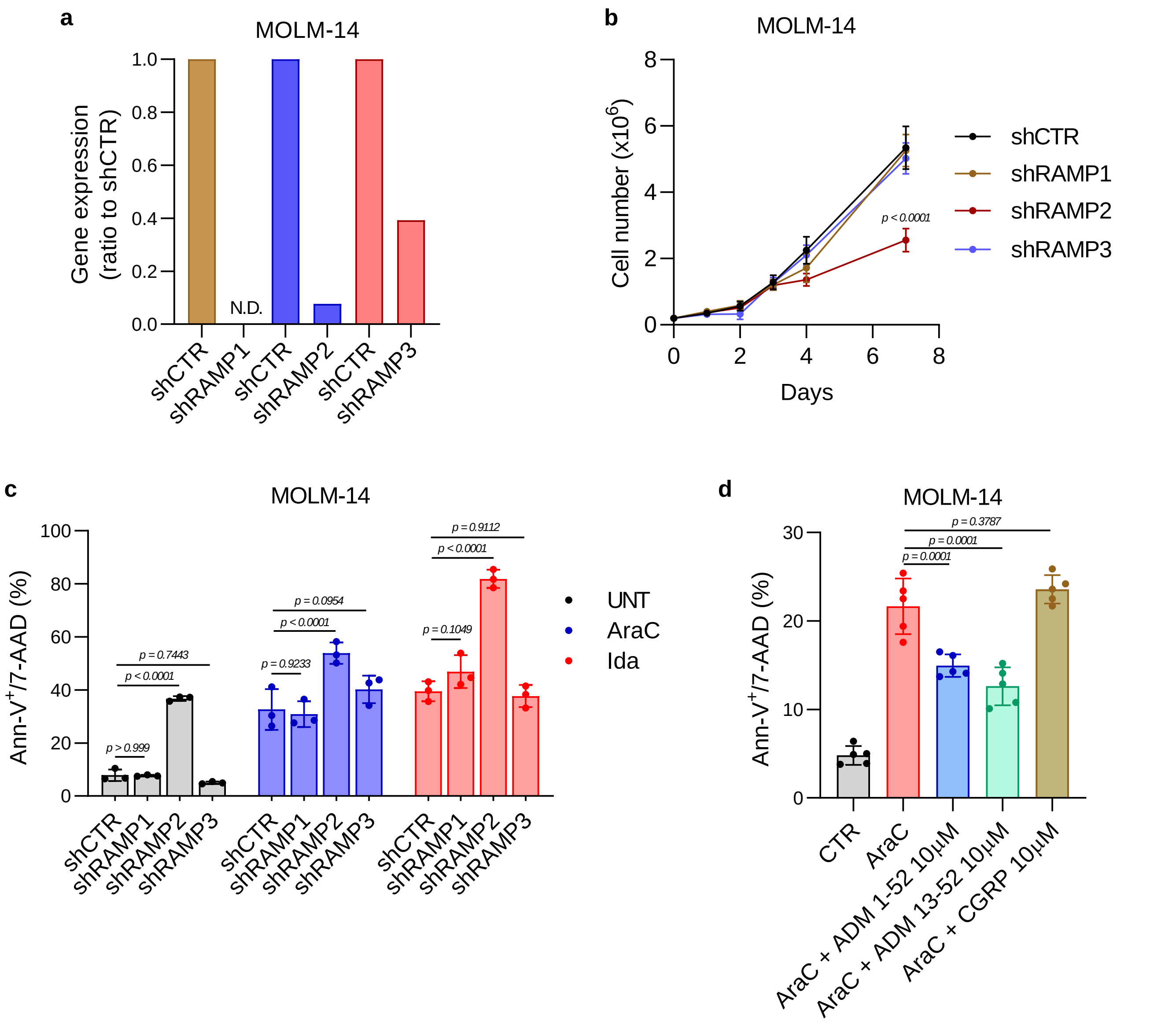

Supplementary Figure 6. RAMP2–mediated ADM signaling drives cell proliferation and resistance to chemotherapeutic agents

a. Gene expression of *RAMP1*, *RAMP2* and *RAMP3* 5 days after induction of shRNA expression with doxycycline (n=1). **b.** Graph shows cell number of MOLM-14 expressing shCTR, shRAMP1, shRAMP2 or shRAMP3. Doxycycline was added at D0 and cell proliferation was followed using trypan blue exclusion (n=4, Two way ANOVA). **c.** Graph shows the percentage of Annexin-V+ or 7-AAD+ cells. Five days after adding doxycycline, MOLM-14 or OCI-AML3 cells expressing inducible shRNAs were treated with AraC or Idarubicin for 48h. Then, the percentage of cell death was monitored using Annexin-V+ and 7-AAD+ markers (n=3, Unpaired t test). **d.** Graph shows the percentage of Annexin-V+ or 7-AAD+ MOLM-14 cells treated 48h with the indicated compound (AraC 1µM) (n=5, Unpaired t test).

Supplementary Figure 7

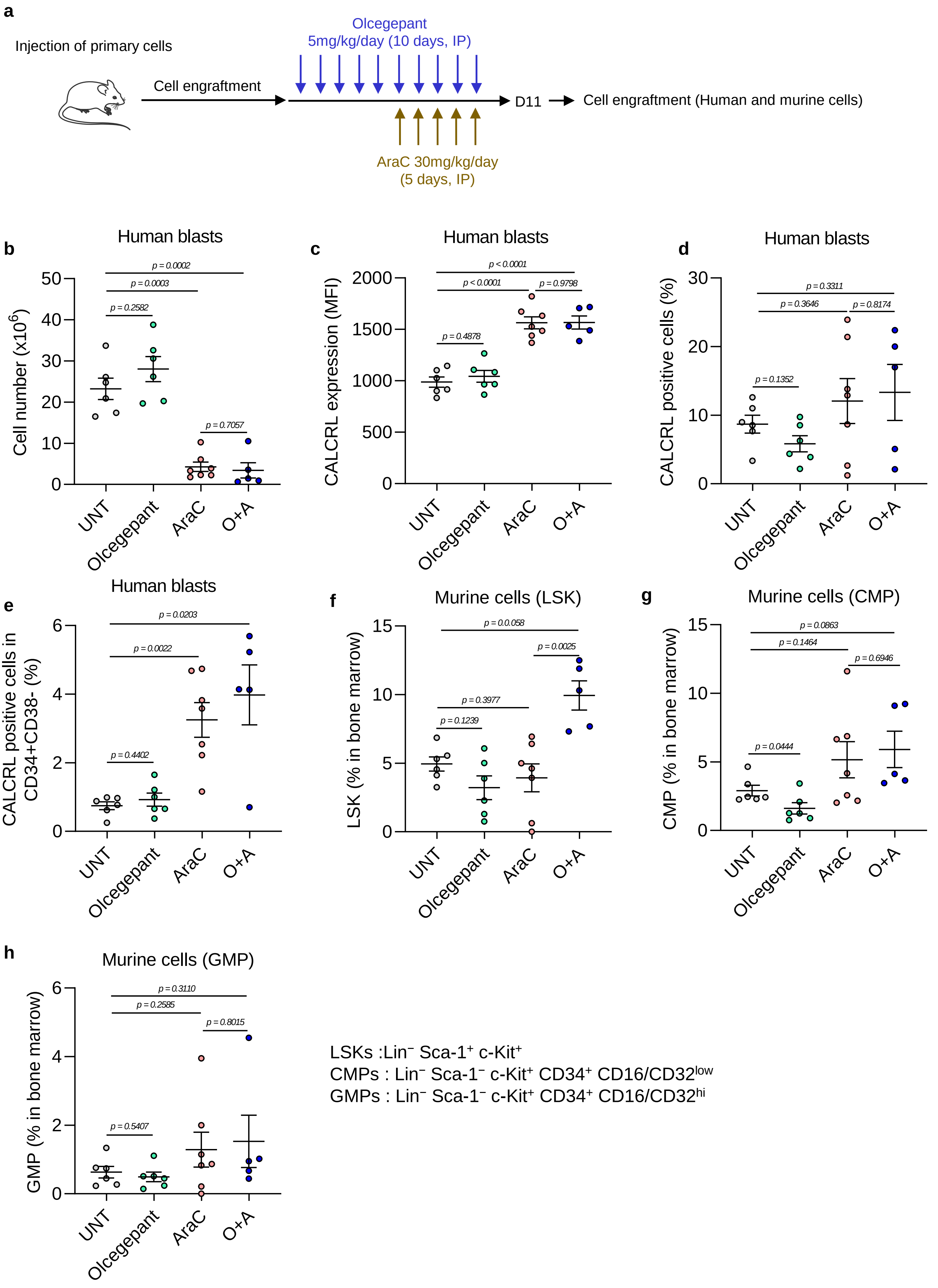

Supplementary Figure 7: *In vivo* inhibition of CGRP signaling in combination with chemotherapy

a. Primary cells were injected in the tail vein of NSG (n=5-7 mice per group) and after leukemic engraftment, mice were intraperitoneally treated with olcegepant (5 mg/kg/d) alone or in combination with AraC (30 mg/kg/d). At day 11, mice were sacrificed and the minimal residual disease was studied. **b.** Leukemic burden (Unpaired t test). **c.** CALCRL expression in human blasts (MFI) (Unpaired t test). **d.** CALCRL positive cells in human blasts (%) (Unpaired t test). **e.** CALCRL positive cells in human CD34+CD38- cells (%) (Unpaired t test). **f.** Impact of treatments on murine LSKs (Unpaired t test). **g.** Impact of treatments on murine CMPs (Unpaired t test). **h.** Impact of treatments on murine GMPs (Unpaired t test).

Supplementary Figure 8

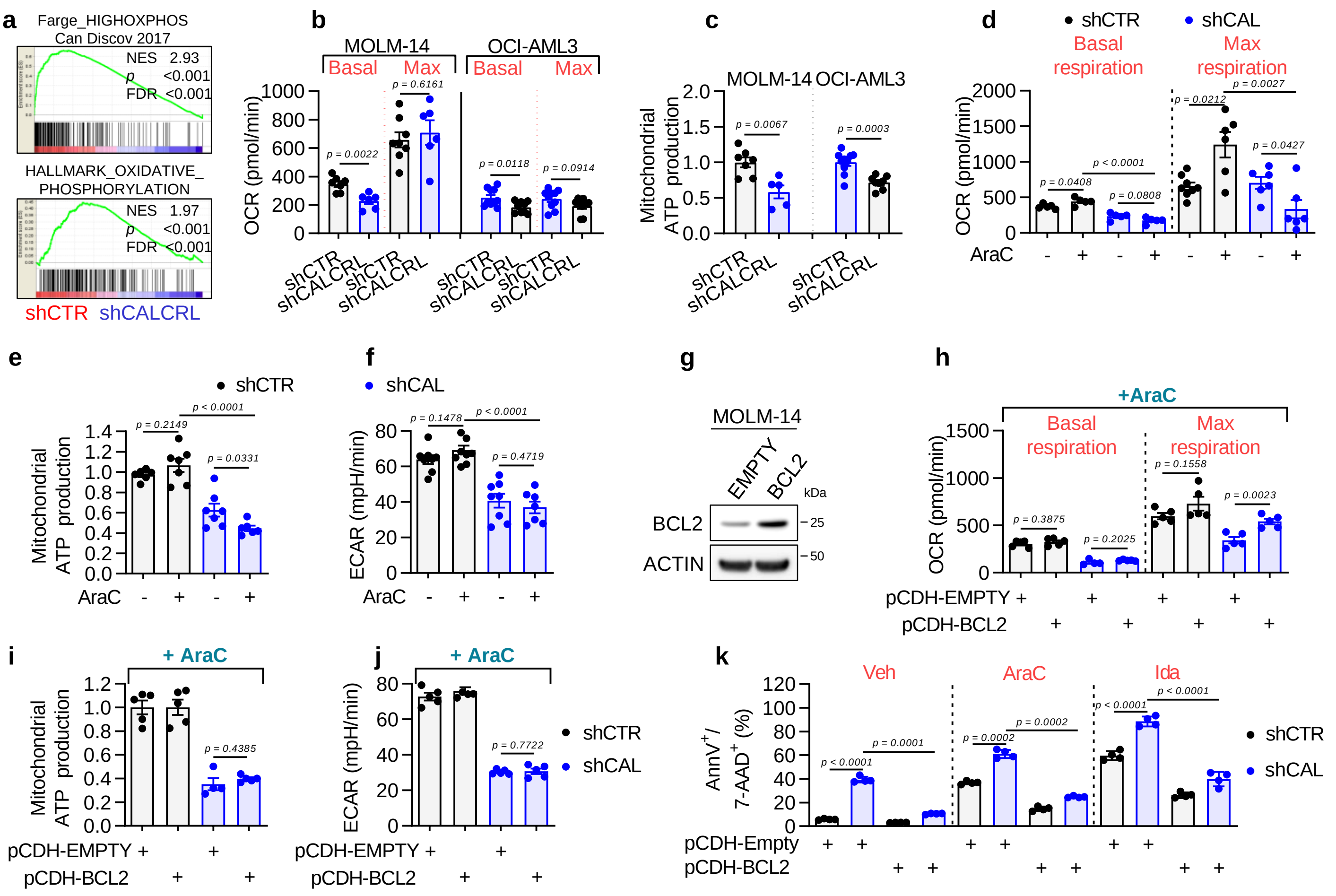

Supplementary Figure 8. Downregulation of CALCRL interferes with OxPHOS status of AML cells in a BCL2-dependent manner.

a. GSEA of Farge_HIGHOXPHOS and HALLMARK_OXIDATIVE_PHOSPHORYLATION gene signatures in transcriptomes of MOLM-14 cells expressing shCTR (red) compared with shCALCRL (blue). Number of permutations = 1000, permutation type = gene_set. Other parameters were left at default values. **b.** OCR was measured by seahorse three days after lentiviral transduction of MOLM-14 or OCI-AML3 cells with a non-targeting shRNA or a shRNA against CALCRL (shCAL#2) (n=6-10, Unpaired t test). **c.** Mitochondrial ATP production was assessed using seahorse (n=5-10, Unpaired t test). **d, e, f** Four days after lentiviral transduction of MOLM-14 cells with shCTR or shCALCRL (shCAL#2), cells were treated with vehicle or 2 µM AraC for 24h and OCR, mitochondrial ATP production and ECAR were measured by seahorse (Unpaired t test). **g.** MOLM-14 transduced with pCDH-EMPTY or pCDH-BCL2 vectors (n=1). **h, i, j** MOLM-14 pCDH-EMPTY or pCDH-BCL2 were transduced with non-targeting shRNA or shRNA targeting CALCRL (CAL#2) for four days. Then cells were treated with 2 µM AraC for 48h before OCR, mitochondrial ATP production and ECAR measurements by seahorse (Unpaired t test). **k.** Cells were exposed to 1µM AraC or 50nM Idarubicin for 48h before analysis of the percentage of AnnV+ or 7-AAD+ cells by flow cytometry (n=4, Unpaired t test).

Supplementary Figure 9

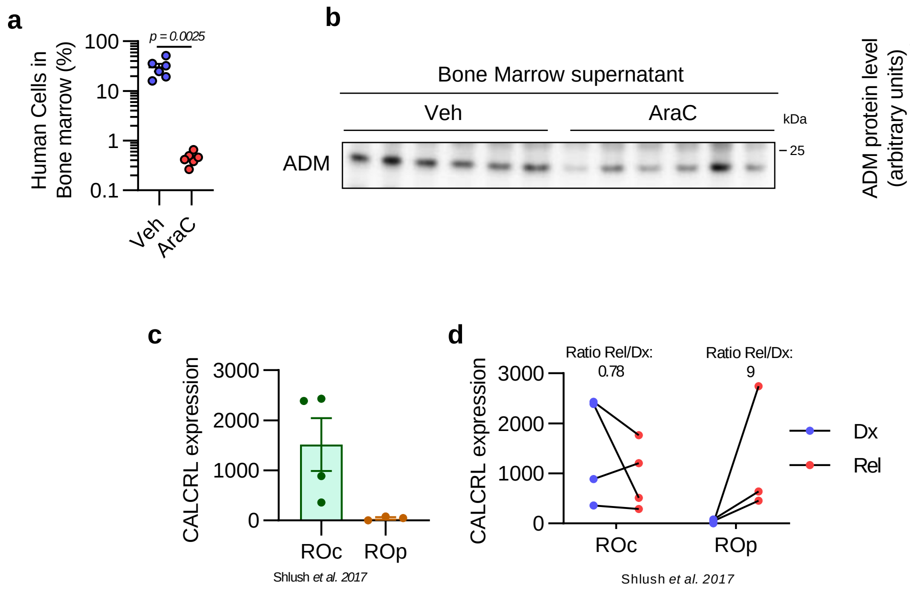

Supplementary Figure 9. CALCRL sustains LSC function of Relapse-initiated Drug Tolerant Cells

a. Percentage of human cells in the murine bone marrow in PBS and AraC-treated mice. **b.** Western-Blot and graph showing the protein expression of ADM in the bone marrow supernatant of xenografted mice treated with PBS or AraC (n=1, Unpaired t test). **c.** CALCRL expression in ROc and ROp patients at the time of diagnosis (Shlush *et al*. 2017). (D) CALCRL expression in ROc and ROp patients at diagnosis or relapse.
