# Supplementary material for: Adrenomedullin-CALCRL axis controls relapse-initiating drug tolerant acute myeloid leukemia cells
Source: Nat Commun. 2021 Jan 18;12:422. doi: 10.1038/s41467-020-20717-9 (PMC7813857; doi:10.1038/s41467-020-20717-9)
Supplement: Supplementary file 3 — Reporting summary [file 41467_2020_20717_MOESM3_ESM.pdf]

nature research

Corresponding author(s): Jean-Emmanuel Sarry

Last updated by author(s): Dec 1, 2020

Reporting Summary

Nature Research wishes to improve the reproducibility of the work that we publish. This form provides structure for consistency and transparency in reporting. For further information on Nature Research policies, seeAuthors & Referees and theEditorial Policy Checklist .

Statistics

For all statistical analyses, confirm that the following items are present in the figure legend, table legend, main text, or Methods section.

n/a	Confirmed	
☐	☒	The exact sample size (*n*) for each experimental group/condition, given as a discrete number and unit of measurement
☐	☒	A statement on whether measurements were taken from distinct samples or whether the same sample was measured repeatedly
☐	☒	The statistical test(s) used AND whether they are one- or two-sided<br>*Only common tests should be described solely by name; describe more complex techniques in the Methods section.*
☒	☐	A description of all covariates tested
☐	☒	A description of any assumptions or corrections, such as tests of normality and adjustment for multiple comparisons
☐	☒	A full description of the statistical parameters including central tendency (e.g. means) or other basic estimates (e.g. regression coefficient) AND variation (e.g. standard deviation) or associated estimates of uncertainty (e.g. confidence intervals)
☐	☒	For null hypothesis testing, the test statistic (e.g. *F*, *t*, *r*) with confidence intervals, effect sizes, degrees of freedom and *P* value noted<br>*Give P values as exact values whenever suitable.*
☒	☐	For Bayesian analysis, information on the choice of priors and Markov chain Monte Carlo settings
☒	☐	For hierarchical and complex designs, identification of the appropriate level for tests and full reporting of outcomes
☒	☐	Estimates of effect sizes (e.g. Cohen's *d*, Pearson's *r*), indicating how they were calculated

Our web collection on statistics for biologists contains articles on many of the points above.

Software and code

Policy information about availability of computer code

Data collection

GeneSys Software v1.6.7.0 (https://www.syngene.com/support/software-downloads/),
CytoExpert Software v2.0 for CytoFLEX v2.0, Beckam Coulter (https://www.beckman.com/coulter-flow-cytometers/cytoflex/cytexpert)
Seahorse XFe24 Wave Desktop v2.6 , Agilent, https://www.agilent.com/en/products/cell-analysis/software-download-for-wave-desktop RRID: SCR_014526,
DIVA Software v6.1.2, BD Biosciences (http://www.bdbiosciences.com/us/instruments/research/software/flow-cytometry-acquisition/bd-facsdiva-software/m/111112/overview)

Data analysis

GSEA Software v4.0 (http://software.broadinstitute.org/gsea/index.jsp)
GeneTool Software v4.3.8.0 (https://www.syngene.com/support/software-downloads/); RRID: SCR_006941
Genomatix Software v3.13 (https://www.genomatix.de/); RRID: SCR_008036
R software v4.0
FlowJo v10.4.2 Software (https://www.flowjo.com/solutions/flowjo/downloads); RRID: SCR_008520
Prism v6.0 and v8.0 Software (https://www.graphpad.com/scientific-software/prism/); RRID: SCR_002798
Seahorse XFe24 Wave Desktop v2.6 (https://www.agilent.com/en/products/cell-analysis/software-download-for-wave-desktop); RRID: SCR_014526
ImageJ bundled with Java v1.8.0_172 (https://imagej.nih.gov/ij/)
OpenComet v1.3.1 software (http://cometbio.org/)

For manuscripts utilizing custom algorithms or software that are central to the research but not yet described in published literature, software must be made available to editors/reviewers. We strongly encourage code deposition in a community repository (e.g. GitHub). See the Nature Research guidelines for submitting code & software for further information.

Data

Policy information about availability of data

All manuscripts must include a data availability statement. This statement should provide the following information, where applicable:
- Accession codes, unique identifiers, or web links for publicly available datasets
- A list of figures that have associated raw data
- A description of any restrictions on data availability

All data are available from the authors upon request. Source data are provided with this paper for Figures 1–7 and Supplementary Figures 1–9. RNA microarray dataset on shControl and shCALCRL MOLM14 cells in independent triplicates were deposited at GEO with accession code GSExxxxx. All publicly accessible transcriptomic databases of AML patients used in this study: GSE30377: Eppert K, Takenaka K, Lechman ER, Waldron L, Nilsson B, van Galen P, Metzeler KH, Poeppl A, Ling V, Beyene J, Canty AJ, Danska JS, Bohlander SK, Buske C, Minden MD, Golub TR, Jurisica I, Ebert BL, Dick JE. (28 August 2011) Stem cell gene expression programs influence clinical outcome in human leukemia. Nat Med, 17(9), 1086-93. GSE14468: Verhaak RG, Wouters BJ, Erpelinck CA, Abbas S, Beverloo HB, Lugthart S, Löwenberg B, Delwel R, Valk PJ. (January 2009) Prediction of molecular subtypes in acute myeloid leukemia based on gene expression profiling. Haematologica, 94(1), 131-4. GSE12417: Metzeler KH, Hummel M, Bloomfield CD, Spiekermann K, Braess J, Sauerland MC, Heinecke A, Radmacher M, Marcucci G, Whitman SP, Maharry K, Paschka P, Larson RA, Berdel WE, Büchner T, Wörmann B, Mansmann U, Hiddemann W, Bohlander SK, Buske C; Cancer and Leukemia Group B.; German AML Cooperative Group. (15 November 2008) An 86-probe-set geneexpression signature predicts survival in cytogenetically normal acute myeloid leukemia. Blood, 112(10), 4193-201. GSE116256: Van Galen P, Hovestadt V, Wadsworth Ii MH, Hughes TK, Griffin GK, Battaglia S, Verga JA, Stephansky J, Pastika TJ, Lombardi Story J, Pinkus GS, Pozdnyakova O, Galinsky I, Stone RM, Graubert TA, Shalek AK, Aster JC, Lane AA, Bernstein BE. Single-cell RNA-seq reveals AML hierarchies relevant to disease progression and immunity. Cell. 2019 Mar 7;176(6):1265-1281. TCGA: The Cancer Genome Atlas Research Network. (30 May 2013) Genomic and epigenomic landscapes of adult de novo acute myeloid leukemia. N Engl J Med, 368(22), 2059-74. Erratum in: N Engl J Med. 2013 Jul 4;369 (1):98. BEATAML: Functional genomic landscape of acute myeloid leukaemia. Nature, 2018 Oct;562(7728):526-531.

Field-specific reporting

Please select the one below that is the best fit for your research. If you are not sure, read the appropriate sections before making your selection.

☒ Life sciences ☐ Behavioural & social sciences ☐ Ecological, evolutionary & environmental sciences

For a reference copy of the document with all sections, see nature.com/documents/nr-reporting-summary-flat.pdf

Life sciences study design

All studies must disclose on these points even when the disclosure is negative.

Sample size	No sample-size calculation was performed. Generally accepted sample sizes were used to allow statistical analysis. Sample size was determined based on standard practices and our previous experience in vitro and in vivo (Sarry et al JCI 2011; Farge et al Cancer Discov 2017). The number of independent experiment and replicates was shown in the corresponding figure legend.
Data exclusions	No data obtained in this study were excluded.
Replication	To verify the reproducibility experimental findings were replicated several times with at least three independent experiments for both in vitro and in vivo conditions.
Randomization	For in vivo studies, mice grafted with primary samples (PDXs) and cell lines (CLDXs) were randomly distributed into cages. While disease established, mice were randomly distributed between arms (AraC vs Placebo) taking into account the peripheral blood engraftment, the weight of mice and their gender.
Blinding	The investigator responsible for in vivo experiments was blinded for the group allocation and at point of analysis, but blinding was not possible during AraC since this compound is opaque and white while the vehicle is transparent. For expression analysis, blinding was not possible to enable orderly loading of the gels. Other experiments presented in this study not required blinding.

Reporting for specific materials, systems and methods

We require information from authors about some types of materials, experimental systems and methods used in many studies. Here, indicate whether each material, system or method listed is relevant to your study. If you are not sure if a list item applies to your research, read the appropriate section before selecting a response.

Materials & experimental systems

n/a	Involved in the study	
☐	☒	Antibodies
☐	☒	Eukaryotic cell lines
☒	☐	Palaeontology
☐	☒	Animals and other organisms
☐	☒	Human research participants
☐	☒	Clinical data

Methods

n/a	Involved in the study	
☒	☐	ChIP-seq
☐	☒	Flow cytometry
☒	☐	MRI-based neuroimaging

Antibodies

Antibodies used

All antibodies used in this study, references and suppliers are described in the manuscript in the section Assessment of Leukemic Engraftment.
anti-CASPASE-3 (CST, Cat#9662; 1/1,000), Anti-ACTIN (Millipore, Cat# MAB1501; 1/10,000), anti-CALCRL (Elabscience, Cat# ESAP13421; 1/1,000), anti-RAD51 (Abcam, Cat# ab133534; 1/1,000), anti-BCL2 (CST, Cat# 2872; 1/1,000), anti-E2F1 (C-20)(Santa Cruz, Cat# sc-193; 1/1,000), anti-CHK1 (Santa Cruz, Cat# sc-8408; 1/1,000), anti-RAMP1 (3B9) (Santa Cruz, Cat# sc-293438; 1/1,000), anti-RAMP2 (B-5) (Santa Cruz, Cat# sc-365240; 1/1,000), anti-RAMP3 (G-1) (Santa Cruz, Cat# sc-365313; 1/1,000), anti-ADM (Thermo Fisher Scientific, Cat# PA5-24927; 1/1,000), anti-CGRP (Abcam, Cat# ab47027; 1/1,000), anti-PARP (Thermo Fisher Scientific, Cat# 44-698G; 1/1,000), anti-alpha/beta-Tubulin (CST, Cat# 2148; 1/1,000).

Validation

All antibodies are from commercial sources and their validation data are available on the manufacturer's website.
anti-CASPASE-3 (CST, Cat#9662) : validation for WB by manufacturer (expression in different cell lines) and specificity provided in the manuscript by the induction of apoptosis. Reactivity for human, mouse, rat, monkey.
anti-ACTIN (Millipore, Cat# MAB1501) : validation for WB by manufacturer. Reactivity for all species..
anti-CALCRL (Elabscience, Cat# ESAP13421) : validation for WB by manufacturer and specificity provided in the manuscript by using shRNA. Reactivity for human.
anti-RAD51 (Abcam, Cat# ab133534) : validation for WB by manufacturer (expression in different cell lines). Reactivity for mouse, rat and human.
anti-BCL2 (CST, Cat# 2872) : validation for WB by manufacturer. Reactivity for human.
anti-E2F1 (C-20) (Santa Cruz, Cat# sc-193): validation for WB by manufacturer and specificity provided in the manuscript by using shRNA. Reactivity for human.
anti-CHK1 (Santa Cruz, Cat# sc-8408): validation for WB by manufacturer (expression in different cell lines). Reactivity for mouse, rat and human.
anti-RAMP1 (3B9) (Santa Cruz, Cat# sc-293438) : validation for WB by manufacturer (expression of human recombinant RAMP1 protein). Reactivity for human.
anti-RAMP2 (B-5) (Santa Cruz, Cat# sc-365240) : validation for WB by manufacturer (expression in different cell lines). Reactivity for mouse, rat and human.
anti-RAMP3 (G-1) (Santa Cruz, Cat# sc-365313) : validation for WB by manufacturer (expression in different cell lines). Reactivity for mouse, rat and human.
anti-ADM (Thermo Fisher Scientific, Cat# PA5-24927) : validation for WB by manufacturer (expression in mouse lung tissue lysate) and specificity provided in the manuscript by using shRNA. Reactivity for mouse and human.
anti-CGRP (Abcam, Cat# ab189786) : validation for WB by manufacturer (expression in PC-12). Reactivity for mouse and human.
anti-PARP (Thermo Fisher Scientific, Cat# 44-698G) : validation for WB by manufacturer (expression in different cell lines) and specificity provided in the manuscript by the induction of apoptosis. Reactivity for mouse, rat and human.
anti-alpha/beta-Tubulin (CST, Cat# 2148) : validation for WB by manufacturer (expression in different cell lines). Reactivity for human, mouse, rat, monkey, zebrafish, bovine.

Eukaryotic cell lines

Policy information about cell lines

Cell line source(s)

All cell lines used in this study are described in the manuscript in the section Assessment of Leukemic Engraftment and were obtained from DSMZ. The U937 cells were obtained from the DSMZ in February 2012 and from the ATCC in January 2014. MV4-11 and HL-60 cells were obtained from the DSMZ in February 2012 and 2016. KG1 cells were obtained from the DSMZ in February 2012 and from the ATCC in March 2013. KG1a cells were obtained from the DSMZ in February 2016. MOLM14 was obtained from Pr. Martin Carroll (University of Pennsylvania, Philadelphia, PA) in 2011.

Authentication

DSMZ and ATCC provide authenticated cell lines by cytochrome C oxidase I gene analysis and short tandem repeat profiling. The names of the used cell lines are authentic and previously published. Nevertheless, all cell lines were sequenced regularly in order to avoid cross-contamination or other mechanisms by STR technique.

Mycoplasma contamination

All cell lines have been routinely tested for Mycoplasma contamination in the laboratory and were negative for contamination.

Commonly misidentified lines
(See ICLAC register)

No commonly misidentified lines were used in this study.

Animals and other organisms

Policy information about studies involving animals; ARRIVE guidelines recommended for reporting animal research

Laboratory animals	NOD/LtSz-SCID/IL-2Rychain null (NSG) mice. Animals were used for transplantation of AML cell lines and primary AML patients cells in accordance with a protocol reviewed and approved by the Institutional Animal Care and Use Committee of Région Midi-Pyrénées (France). NSG mice were produced at the Genotoul Anexplo platform at Toulouse (France) using breeders obtained from Charles River Laboratories. Mice were housed in sterile conditions using HEPA-filtered microisolators and fed with irradiated food and sterile water. 8 weeks old male and female mice (1:1) were sublethally treated with busulfan (20 mg/kg) 24 hours before injection of AML cells.
Wild animals	The study did not involve wild animals.
Field-collected samples	The study did not involve samples collected from field.
Ethics oversight	This study was approved by the Institutional Animal Care and Use Committee of Région Midi- Pyrénées (France).

Note that full information on the approval of the study protocol must also be provided in the manuscript.

Human research participants

Policy information about studies involving human research participants

Population characteristics	Population characteristics correspond to adult AML patients from our clinical site [Toulouse University Hospital (TUH), Toulouse, France]. Detailed information about age, gender, karyotypes, mutations, received treatments are available in Supplementary Table S5.
Recruitment	Primary AML patient cells have been collected during routine diagnostic procedures at the TUH after informed consent.
Ethics oversight	Primary AML patient cells from TUH have been stored at the HIMIP collection (BB-0033-00060). According to the French law, HIMIP collections has been declared to the Ministry of Higher Education and Research (DC 2008-307 collection 1) and obtained a transfer agreement (AC 2008-129) after approbation by the "Comité de Protection des Personnes Sud-Ouest et Outremer II" (ethical committee).<br>Detailed information on the AML tissue microarray cohort has been published previously in Angenendt et al, Leukemia 2019. (DOI: 10.1038/s41375-019-0505-x). Studies on the tissue microarray have been approved by the institutional review board of the University of Münster.

Note that full information on the approval of the study protocol must also be provided in the manuscript.

Clinical data

Policy information about clinical studies

All manuscripts should comply with the ICMJEguidelines for publication of clinical research and a completedCONSORT checklist must be included with all submissions.

Clinical trial registration	N/A
Study protocol	N/A
Data collection	N/A
Outcomes	N/A

Flow Cytometry

Plots

Confirm that:

- [x] The axis labels state the marker and fluorochrome used (e.g. CD4-FITC).
- [x] The axis scales are clearly visible. Include numbers along axes only for bottom left plot of group (a 'group' is an analysis of identical markers).
- [x] All plots are contour plots with outliers or pseudocolor plots.
- [x] A numerical value for number of cells or percentage (with statistics) is provided.

Methodology

Sample preparation	AML cells in culture or from murine bone marrow and spleen were spun down and processed for staining as described in the Material and Methods section.

Instrument	CytoFLEX flow cytometer (Beckman Coulter).
Software	FlowJo v10.4.2.<br>CytoExpert Software for CytoFLEX 2.0, Beckam Coulter
Cell population abundance	FACS analysis of post-sorting fractions was always performed to control its purity.
Gating strategy	Single cells were selected by FSC/SSC, FSC-H/FSC-A and SSC-H/FSC-A gates. Human viable blasts from bone marrow and spleen of AML-engrafted mice were selected based on the expression of hCD45, hCD44 and CD33.

☒ Tick this box to confirm that a figure exemplifying the gating strategy is provided in the Supplementary Information.
